# Avionics Module Fault Diagnosis Algorithm Based on Hybrid Attention Adaptive Multi-Scale Temporal Convolution Network

**DOI:** 10.3390/e26070550

**Published:** 2024-06-27

**Authors:** Qiliang Du, Mingde Sheng, Lubin Yu, Zhenwei Zhou, Lianfang Tian, Shilie He

**Affiliations:** 1 School of Automation Science and Engineering, South China University of Technology, Guangzhou 510641, China; 2 Guangdong Engineering Research Center of Cloud-Edge-End Collaboration Technology for Smart City, Guangzhou 510641, China; 3 The Fifth Electronics Research Institute of Ministry of Industry and Information Technology, Guangzhou 511370, China

**Keywords:** avionics module, fault diagnosis, adaptive convolution, attention mechanism, information entropy

## Abstract

Since the reliability of the avionics module is crucial for aircraft safety, the fault diagnosis and health management of this module are particularly significant. While deep learning-based prognostics and health management (PHM) methods exhibit highly accurate fault diagnosis, they have disadvantages such as inefficient data feature extraction and insufficient generalization capability, as well as a lack of avionics module fault data. Consequently, this study first employs fault injection to simulate various fault types of the avionics module and performs data enhancement to construct the P2020 communications processor fault dataset. Subsequently, a multichannel fault diagnosis method, the Hybrid Attention Adaptive Multi-scale Temporal Convolution Network (HAAMTCN) for the integrated functional circuit module of the avionics module, is proposed, which adaptively constructs the optimal size of the convolutional kernel to efficiently extract features of avionics module fault signals with large information entropy. Further, the combined use of the Interaction Channel Attention (ICA) module and the Hierarchical Block Temporal Attention (HBTA) module results in the HAAMTCN to pay more attention to the critical information in the channel dimension and time step dimension. The experimental results show that the HAAMTCN achieves an accuracy of 99.64% in the avionics module fault classification task which proves our method achieves better performance in comparison with existing methods.

## 1. Introduction

The avionics system refers to the electronic equipment required to support various functions of an aircraft. It is an essential component of the modern aircraft, handling flight control, navigation, communications, surveillance, and a wide range of other critical operations [1]. With the rapid development of aviation technology, as the core of the avionics system, the avionics module is becoming more complex and integrated, and the demand for its improved performance, reliability, and safety is also increasing [2].

Fault prediction and health management are vital means of boosting system availability and maintenance efficiency, and for minimizing lifecycle costs. It is difficult to conduct fault diagnosis of avionics systems with more mature health degradation models; additionally, the lack of specific health data acquisition sensors prevents the health management system from obtaining abundant data to enable data-driven monitoring and fault diagnosis [3]. Therefore, timely and accurate fault diagnosis of the avionics module, as well as implementing effective measures to repair the detected fault and thus maintain the operation of the module, is of great significance to ensure the safe and reliable operation of aircraft [2].

The various operation states and numerous failure modes of avionics modules lead to a broad distribution of fault signals with significant uncertainty, which are typical large-entropy signals. Considering the traditional methods, the equivalent circuit method is difficult to model, the signal-based Fast Fourier Transform (FFT) and Wavelet Transform methods are susceptible to noise interference, and the machine learning-based Support Vector Machine (SVM) and K-Nearest Neighbor (KNN) algorithm have low accuracy [4,5]. In conclusion, traditional methods perform inadequately in dealing with large entropy failure signals of the avionics module.

Because of the dramatic increase in the volume of recorded data in recent years, deep learning-based methods, due to their impressive data exploitation capability, have shown remarkable advantages and wide applicability with large-entropy signals in the field of fault diagnosis. However, in avionics systems, data acquisition faces several challenges, such as data scarcity, security constraints, and complex system architectures. Additionally, the avionics module contains a wide variety of electronic components with diverse properties, creating additional challenges in the training and deployment of deep learning models.

To address the aforementioned limitations, we first simulate several fault states of the functional circuit module of the P2020 communications processor by using the fault injection technique; subsequently, we propose a multichannel fault diagnosis method, HAAMTCN, for the large-entropy fault signals of the avionics module, which utilizes Adaptive Multi-scale Temporal Convolution (AMTC) module adaptively and selects the convolution kernel size based on frequency domain information to fully extract temporal features. Meanwhile, it combines the Interaction Channel Attention (ICA) module and the Hierarchical Block Temporal Attention (HBAM) module as a hybrid attention mechanism to focus the whole model on more valuable information. The ICA module groups the channel dimensions into multiple sub-features and realizes the modulation between different channels through channel interactions. The HBTA module considers both time-step localized associations as well as long-time dependencies. This study makes the following contributions:

1. The Adaptive Multi-scale Temporal Convolution (AMTC) module is proposed, which utilizes the FFT to search for the frequency of the channel with the largest contribution, and adaptively constructs the optimal multi-scale receptive field at an extremely rapid speed, to realize the comprehensive extraction of the features of the time series data. Additionally, residual concatenation is adopted to improve the accuracy and stability of the network to enable stronger feature extraction capability for time series data.
2. The Interaction Channel Attention (ICA) module is presented, which reshapes some channels into batch dimensions and groups the channel dimensions into multiple sub-features to better preserve the information of each channel and reduce the computational overhead. Meanwhile, the regulation between different channels is realized through channel interaction, enabling the model to dynamically learn and adjust the weight of different channels.
3. The Hierarchical Block Temporal Attention (HBTA) module is proposed, which applies the multi-head attention mechanism to the raw feature maps in blocks to effectively extract the local information while reducing the computational complexity and simultaneously performing the downsampling operation on the raw feature maps to expand the scope of multi-head attention to obtain sufficient global information. Finally, by combining the information at different hierarchical blocks, the relationship between time steps of sequential data can be effectively captured, which enhances the expressive power of the model.
4. Several fault states of the functional circuit module of the integrated modular avionics (IMA) system are simulated by using the fault injection technique, and each abnormal state can be equated to the actual situation of a certain function degradation of the electronic module; this leads to the sampling and production of the dataset of avionics module faults. This dataset can provide a database for subsequent avionics module failure analysis.

## 2. Related Work

Presently, avionics module fault diagnosis methods are mainly categorized into model-based methods, signal-based methods, and data-driven methods.

### 2.1. Model-Based and Signal-Based Approaches

Model-based fault diagnosis methods are based on mathematical and physical models that realize the effective identification and assessment of system faults by accurately describing the characteristics of the diagnosed objects and verifying them against actual data. A previous study [6] detected chip faults based on a cellular automata array through compressed test vector coding combined with byte error correction coding techniques. In another study [7], a dynamic analysis method was proposed for state-correlated IMA fault recovery that examined the system state through the analysis of its dynamic behavior. A fault time propagation diagram [8] that provided a diagnostic basis for system faults was established through system analysis for IMA systems. Furthermore, other studies [9] have utilized fault propagation diagrams for the effective fault diagnosis of complex electronic systems with multichannel diagnostic information. While model-based fault diagnosis methods provide us with an effective means to analyze and address system faults, there are some obvious drawbacks and limitations when they are applied to avionics modules. First, avionics systems are structurally complex; therefore, modeling them is significantly challenging. Furthermore, many model-based methods are post hoc diagnostic, i.e., they can only analyze and diagnose faults after they have occurred.

Therefore, signal processing techniques are being increasingly used to meet the needs of fault diagnosis more efficiently and directly. Additionally, these approaches are more intuitive and flexible as compared to model-based methods; they also require less complex modeling processes, thus improving the efficiency and accuracy of fault diagnosis. Some researchers [10] reviewed the commonly utilized signal processing algorithms in electronic equipment fault diagnosis, including Time Synchronous Averaging, FFT, and Envelope Spectrum Analysis, and discussed the applicable scenarios of each method in depth. Additionally, a previous study [11] proposed a multiparameter evaluation method based on confidence value and Mahalanobis distance for the health assessment of complex programming logic device (CPLD) functional modules in an avionics system. While they have a wide range of applications in the field of fault diagnosis, these methods suffer from several problems and challenges. For example, in the face of noise-laden fault signals, the recognition ability of some signal processing methods may be insufficient. The processing of multichannel data is also a challenge for signal processing methods. In avionics systems, multiple sensors may simultaneously generate a large volume of data; therefore, determining how to extract key feature information from such data by signal processing methods needs to be investigated by future researchers.

### 2.2. Data-Driven Approach

Data-driven methods include traditional machine learning methods and deep learning methods.

The core idea of traditional machine learning methods is using abundant monitoring data to construct data-driven models, further extracting key fault features from massive data to realize the intelligent diagnosis of a system’s status. A previous study [12] used the SVM technique to achieve the effective diagnosis of single and multiple faults in induction motors by measuring stator current and vibration signals. Another study [13] employed fault injection to simulate faults in the avionics system; the authors utilized the Back Propagation (BP) neural network for deep data mining, ultimately constructing an effective fault diagnosis algorithm. This approach reduces the dependence on expert knowledge and allows the diagnostic process to be more automatic and intelligent. However, machine learning methods, with their relatively simple model structures, are restricted in their fitting ability when processing high-dimensional data and complex circuit function correlation functions. Moreover, researchers tend to apply different machine learning models to different scenarios.

With the rapid upgrading of hardware equipment and progress of data processing technology, deep learning technology is gradually dominating the field of fault diagnosis and becoming a hot spot and mainstream direction for research. In one study [14], the soft failure signals of the avionics module was modeled employing fault injection; this significantly enhanced the accuracy of avionics system fault diagnosis based on Residual Neural Network (Res Net). A previous study [15] proposed an innovative method to stack transform the original signal to eliminate the effect of manual feature extraction; meanwhile, the researchers constructed a two-dimensional convolutional neural network model based on LeNet-5, that achieved a remarkable performance enhancement in fault diagnosis as compared to that by the traditional methods. Another study [16] proposed a method based on a time series encoder that consisted of a convolutional neural network and innovatively incorporated a convolutional attention mechanism before the output; this method was capable of accurately extracting fault-related information from longer variable length data. Another study [17] proposed a Multivariate Long Short-Term Memory Fully Convolutional Network (MLSTM-FCN) model for multichannel inputs by combining a full convolution module and a Long Short-Term Memory (LSTM) module as a feature extractor, further introducing a Squeeze-and-Excitation Block (SE) Channel attention mechanism module; this model exhibited excellent performance when dealing with multivariate time series tasks. In another study [4], researchers designed a multi-branch residual module comprising dilated convolution; this module was capable of effectively extracting multi-scale features in avionics equipment signals, thus providing richer and more accurate feature information for fault diagnosis. In the meantime, an innovative multiresolution hypergraph neural network algorithm [18] that reveals higher-order complex associations among samples and digs deeper into the underlying structures in the data by constructing and integrating hypergraph structures at multiple resolutions was proposed. In one study [19], the vibration signals generated by faults were converted into time-frequency images using Wavelet Transform methods; subsequently, a convolutional neural network-based fault diagnosis method was designed using these feature images. In some previous studies [20,21,22], a method for constructing a deep neural network was proposed, in which the network design was interpretable, and the network convolution parameters and fault characteristics corresponding to the fault were obtained through learning to exhibit excellent performance. However, regarding the electronic systems domain, research on deep learning applications is lagging behind to some extent as compared to that for the mechanical equipment domain due to factors such as the challenging nature of data acquisition and variability of electronic components. Nonetheless, with the continuous improvement in technology and further research, the potential of deep learning in the application of electronic system fault diagnosis remains enormous.

## 3. Methodology

### 3.1. HAAMTCN Structure

For avionics module fault signals, which are high-information entropy signals, it is necessary to pay attention to the correlation of the information between the channels, and at the same time, the speed of the degradation process varies for different fault types as well as for different channel signals. Therefore, multiple convolution kernels of different sizes are used to extract feature information from multiple scales. In the meanwhile, current state-of-the-art methods often require extensive tuning of hyperparameters to enhance the quality of the extracted features for better extraction of data features. This often requires a significant amount of time. So, for the avionics module diagnostic task, we propose the HAAMTCN structure.

**Figure 1:**
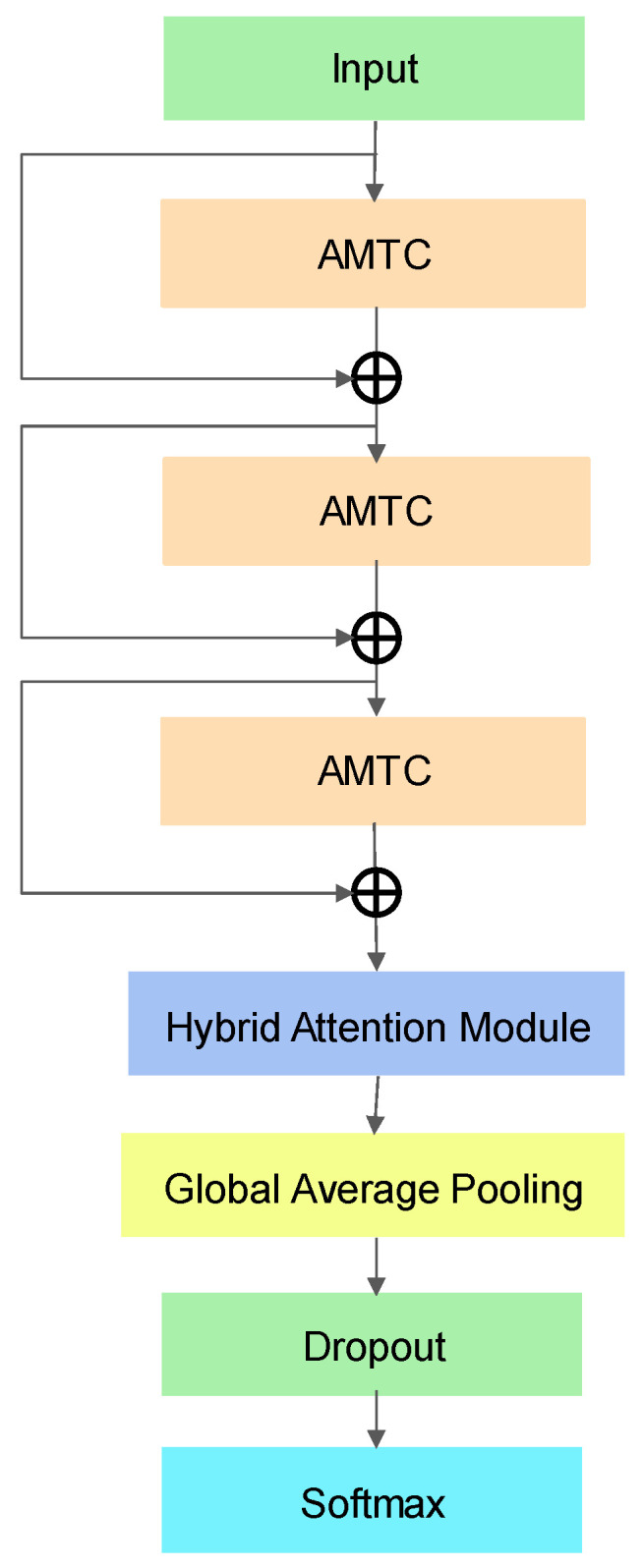
HAAMTCN structure.

The network architecture proposed in this study is illustrated in Figure 1, where the time series dataset of different fault types obtained by injecting anomalies is used as inputs that pass through three Adaptive Multi-scale Temporal Convolution (AMTC) modules and use the residual network structure. Problems encountered during the training of deep neural networks such as gradient vanishing and training difficulties are solved by residual connection that also improves the expressive and generalization abilities of the network, in which the residual connection branch passes through a 1 × 1 one-dimensional convolutional layer and Batch Normalization layer.

**Figure 2:**
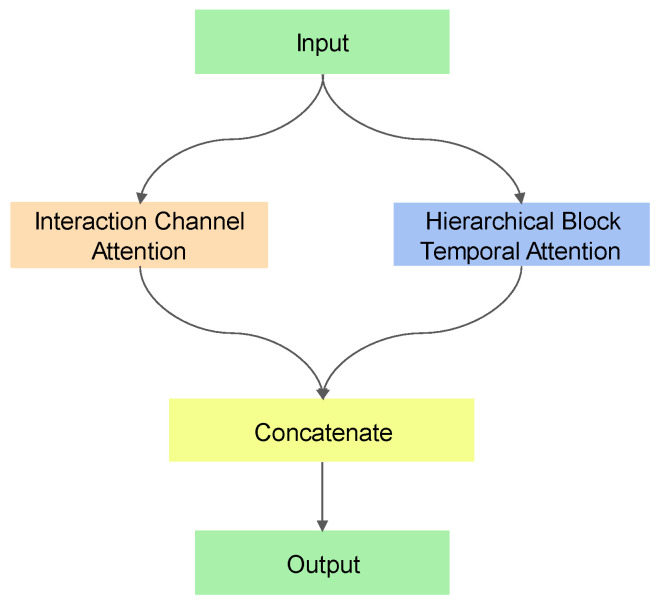
Hybrid Attention module.

The obtained feature maps are input to the Hybrid Attention module (Figure 2) that comprises Interaction Channel Attention (ICA) module and Hierarchical Block Temporal Attention (HBTA) module. Combining these modules to process the input data and splicing the processed results together to extract important feature information from the input data leads to enhancing the model’s attention to the input data and feature representation. The result is passed through the Global Average Pooling and Dropout layer. The Global Average Pooling averages the features on the whole feature map to capture the global information of the whole feature map, thus enabling the model to better learn the most important features. It also reduces the number of parameters and mitigates model complexity, thus reducing the risk of overfitting. Finally, the different types of failure modes are categorized through SoftMax.

On the one hand, the model adaptively selects the convolutional kernel size and extracts sufficient feature information through a three-layer AMTC module with residual connection. Due to its strong feature extraction capability advantage, it is extremely suited for processing large information entropy signals such as avionics fault signals. On the other hand, Interaction Channel Attention and Hierarchical Block Temporal Attention are applied through the Hybrid Attention module to train the model to focus on the critical information of the feature map; eventually, the Global Average Pooling and Dropout layers reduce the parameter acceleration algorithm and minimize the risk of overfitting, which has a remarkable effect on equipping the model to distinguish different types of faults in the avionics module.

### 3.2. Adaptive Multi-Scale Temporal Convolution Module

The Adaptive Multi-scale Temporal Convolution (AMTC) module proposed in this study exploits the advantages of strong convolutional feature extraction capability, as well as adaptive selection of convolution kernel size when dealing with the large information entropy data. In this study, the spectrum for each channel of the fault signal is first obtained according to the FFT, and the approximate optimal receptive field is determined based on the frequency corresponding to the maximum amplitude of each channel. A one-dimensional convolutional layer with a kernel size of 3 × 1 is introduced in the initial stage to match the optimal receptive fields corresponding to the largest number of channels and refine finer features. Subsequently, these features are fed into a multichannel one-dimensional convolution process, where the convolution kernels are of sizes 1 × 1, 9 × 1, 13 × 1, and 27 × 1—each of which corresponds to an optimal receptive field. These convolution results are spliced with the input features processed by maximum pooling to form a completely new feature map. Thereafter, this feature map is again subjected to multichannel parallel one-dimensional convolutional processing for secondary feature extraction, this time with convolutional kernel sizes of 1 × 1, 3 × 1, 5 × 1, and 7 × 1. Smaller convolutional kernel sizes are chosen for better identification of local patterns and features. Each splicing operation is immediately followed by a Batch Normalization layer and Rectified Linear Unit layer, the combination of which not only accelerates the training process but also makes the whole training more robust.

**Figure 3:**
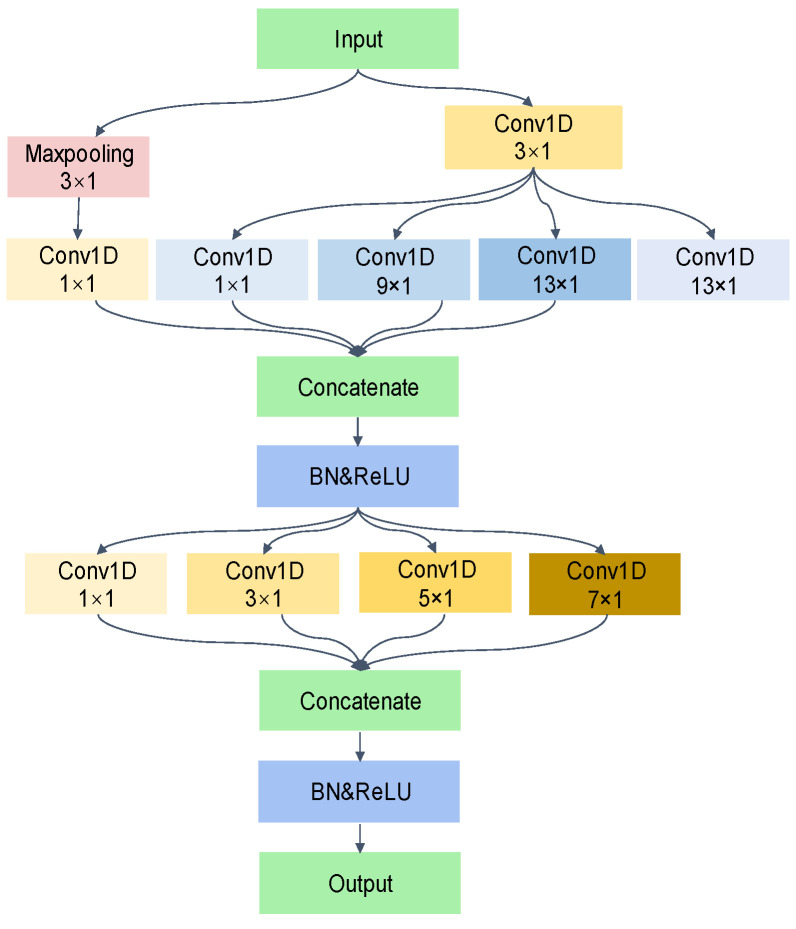
AMTC module.

Through this design, the AMTC module can not only adaptively select the appropriate convolutional kernel size to approximate the optimal receptive field, which significantly reduces the computational overhead regarding selecting suitable convolutional kernels, but at the same time, the utilization of the multichannel parallel convolutional strategy also dramatically improves the training efficiency of the model. The AMTC module is illustrated in Figure 3.

For the convolution before the first splicing, a different convolutional kernel size is selected for each branch to obtain more sensory fields, and the output of the multi-scale convolution is spliced in the channel dimension. The output obtained by splicing can be expressed as follows:

$$x_{concat}=Concat\left(x_{out}^{1},x_{out}^{2},\ldots ,x_{out}^{n},x_{pooling}\right)$$

where $x_{out}^{i}$ represents the output of the branch I, that can be expressed as

$$x_{out}^{i}=f_{1}^{i}f_{0}(x_{i})$$

where $f_{0}$ denotes the 3 × 1 convolution, $f_{1}^{i}$ denotes the convolutional layer corresponding to branch *i*;

$x_{pooling}$ represents the pooling channel and can be expressed as follows:

$$x_{pooling}=f_{2}\left(Maxpooling\left(x_{i}\right)\right)$$

where $f_{2}$ is the 1 × 1 convolution operation.

**Figure 4:**
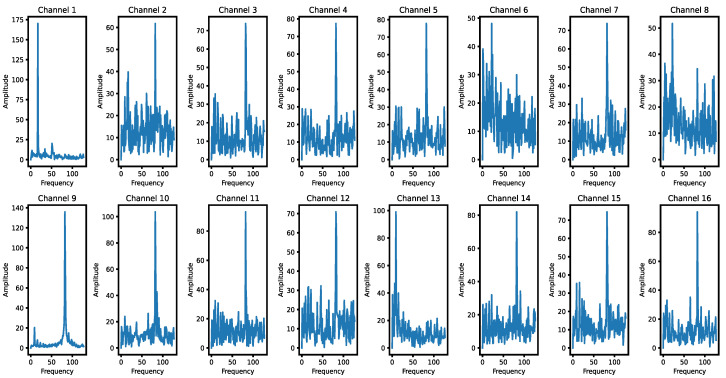
Frequency domain characteristics of each channel in fault class 1.

Based on the FFT, the amplitude of each channel of the fault signal at different frequencies can be obtained. The amplitude represents the intensity of each frequency component in the signal spectrum, and the frequency component with a larger amplitude corresponds to the signal with larger energy concentrated at that frequency. The period with the largest contribution from each channel is calculated based on the frequency with the largest amplitude; thus, the appropriate convolutional kernel size is adaptively selected to roughly obtain the optimal receptive field that greatly reduces the computational overhead required to sample various sizes of convolutional kernels. The frequency domain characteristics of the individual channels of fault class 1 are shown in Figure 4.

For periodic time series data, the optimal receptive field should be able to cover a full cycle so that the network can capture the key features in the periodic pattern. If the receptive field is too small to cover the complete cycle, it will result in the network failing to learn periodic features effectively. In contrast, if the sensory field is too large, it will contain information from multiple cycles, leading to imprecisely learned features. Therefore, we chose a period of 1–2 times of the period corresponding to the maximum frequency of the contribution as the approximate optimal receptive field.

The optimal receptive field is formulated as follows:

$$l_{k}=l_{k-1}+\left(\left(f_{k}-1\right)*\prod_{i=1}^{k-1}s_{i}\right)$$

$l_{k}$ denotes the receptive field of layer *k*, $l_{k-1}$ denotes the receptive field of the layer *k* − 1, $f_{k}$ denotes the size of the convolution kernel of layer *k*, and $s_{i}$ denotes the step size of layer *i*.

### 3.3. Interaction Channel Attention Module

In this section, we present the ICA (Interaction Channel Attention) module for time series data. This module incorporates both 1 × 1 and 3 × 1 branches to fulfill inter-channel information interaction and cross-channel feature extraction. This design not only facilitates the capture of deep correlations between channels but also effectively extends the receptive field of the network, thus offering enhanced characterization capabilities when processing complex time series patterns. For a multichannel time series, $X=(x_{1},x_{2},\ldots ,x_{n})\in {\mathbb{R}}^{\left(c\times n\right)}$, where $x_{i}$ denotes the vector of time step *i*, c denotes the number of channels, and n denotes the number of time steps, that is, the length of the sequence. The input is first divided into g groups through the group layer, reshaped into batch dimensions, and finally grouped into multiple sub-features of the channel dimensions. Through the Global Average Pooling process and in conjunction with the Group Normalization layer, the attentional weight within each channel group is computed and applied to the channel features within the group to achieve the weighting of different channels within the group. Finally, the weighted in-group features are recombined into the final output; this ensures that the model retains key information while effectively reducing computational overhead, thus demonstrating superior performance and efficiency when dealing with large-scale time series data.

**Figure 5:**
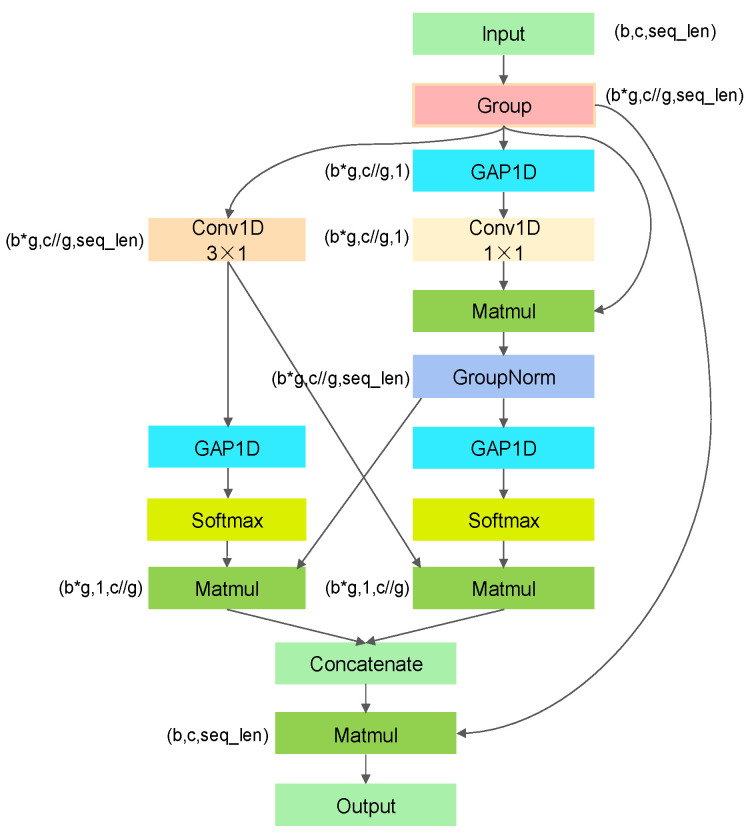
ICA module. Here, b denotes the batch size, c denotes the number of channels, seq_len denotes the sequence length, and g denotes the number of groups. During the dimensional transformation, * denotes multiplication and // denotes division.

Overall, this channel attention mechanism realizes the proposed network’s adaptive selection and weighting of channel information by dynamically adjusting the importance weight of each channel, thus promoting the model’s characterization ability and performance. Through channel interactions, the network can exploit inter-channel correlations more effectively and selectively strengthen or weaken the influence of specific channels according to the task requirements, thus improving the behavior and generalization of the model. The structure of the ICA mechanism is illustrated in Figure 5.

### 3.4. Hierarchical Block Temporal Attention Module

Self-attention is a mechanism for a sequence module that dynamically assigns different attentional weights to elements at different locations when processing sequence data, thus allowing the sequence module to better capture the relationships between different parts of the sequence. However, its computational complexity is high, and it has limitations for capturing long-distance dependencies between different positions in a sequence, and it lacks positional information itself.

To solve the above problem, Our Hierarchical Block Temporal Attention (HBTA) module acts on time series data by assigning different levels of attention to information at different time steps. The HBTA module divides the raw multichannel time series feature maps into multiple non-overlapping sub-feature maps along the time-step dimension using a suitably sized window, and the surrounding deficiencies are filled using 0 values. A multi-head attention mechanism is applied to each sub-feature map, and then the results are stitched together by the location of the blocks, avoiding interference from too distant time points while reducing the computational effort. At the same time, the original feature maps are downsampled multiple times using a large step-length one-dimensional convolution to shorten the length of the time series and keep the number of channels constant. The same number of blocks as in the previous layer are adopted, and then the relationship between time steps is extracted by using the multi-head attention mechanism for the sub-feature maps after the blocking. Finally, upsampling from bottom to top using transposed convolution expands its size to be the same as the result of the previous layer’s self-attention computation, with the number of channels unchanged, and then sums up with the result of the previous layer’s self-attention on an element-by-element basis up to the uppermost layer. 

**Figure 6:**
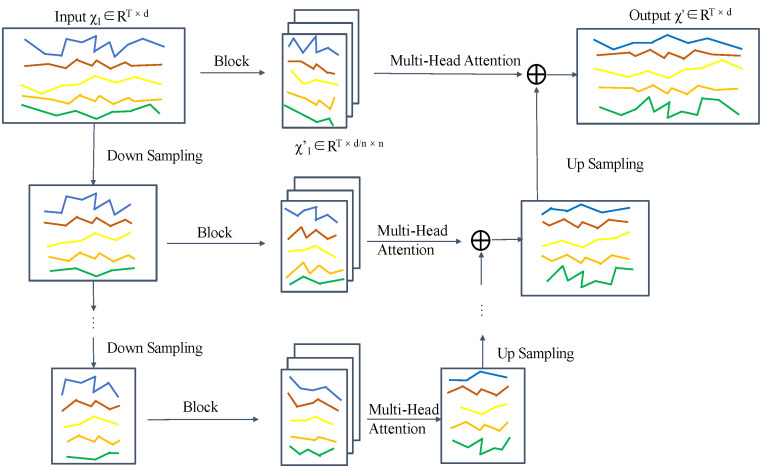
HBTA module. Lines of different colors denote time series of different channels.

In this way, a larger range of time point information can be obtained, which is ultimately equivalent to the self-attentive computation of all time point information within the entire time series feature map, while the computational effort is greatly reduced. Using the multi-attention mechanism through the way of blocking in different positions can include the features in the time point more comprehensively, obtain the multi-angle time-step self-attention information, and improve the accuracy and comprehensiveness of the time-step self-attention. The structure of the HBTA module proposed in this paper is shown in Figure 6.

For the input feature map X, multiply each of the three feature matrices $W^{Q}$, $W^{K}$, $W^{V}$:

$$\begin{array}{c} Q=XW^{Q}\\ K=XW^{K}\\ V=XW^{V} \end{array}$$

Using *Q*, *K*, and *V*, the final output result matrix of the Attention layer is calculated:

$$Attention\left(Q,K,V\right)=soft\max\left(\frac{QK^{T}}{\sqrt{d_{k}}}\right)V$$

$$d_{k}=d_{hid}//n$$

where $d_{hid}$ is the size of the hidden layer of the multi-head attention mechanism and n is the number of multi-head attention mechanism heads.

The original feature map is downsampled using one-dimensional convolution, and the relationship between the input time step $L_{in}$ and the output time step $L_{out}$ is

$$L_{out}=\frac{\left(L_{in}+2\times Padding-KernelSize\right)}{Stride}+1$$

The upsampling operation is performed on the generated feature map using transposed convolution, and the relationship between the input time step $L_{in}^{\prime }$ and the output time step $L_{out}^{\prime }$ is

$$L_{out}^{\prime }=\left(L_{in}^{\prime }-1\right)\times Stride-2\times Padding+KernelSize$$

where *Padding* denotes the padding length, *KernelSize* denotes the one-dimensional convolutional kernel size, and Stride denotes the convolutional kernel move step.

The HBTA module effectively captures the relationship between the time steps of sequence data. Capturing local temporal correlations independent of the long-range dependencies of the entire sequence by applying multi-head attention at the sub-block level simultaneously reduces the computational complexity of the multi-head attention mechanism and enhances the robustness of the model. Local correlations in the sequence can be better captured by downsampling and applying the multi-attention mechanism on shorter sequences without the limitation of the length of the original sequence, and the feature information in the sequence can be extracted more efficiently. Upsampling the results of lower-level attention calculations and splicing them with the results of upper-level attention can enable the model to take into account different levels of information at the same time, enhancing the expressive power of the model and enabling it to better understand the structure and characteristics of the entire time series.

## 4. Experiment

### 4.1. Evaluation Metrics

Accuracy, precision, recall, and F1-Score were chosen as the evaluation metrics and were calculated as follows:

$$Accuracy=\frac{TP+TN}{TP+FP+FN+TN}$$

$$Precision=\frac{TP}{TP+FP}$$

$$Recall=\frac{TP}{TP+FN}$$

$$F1-Score=\frac{2Precision\times Recall}{Precision+Recall}$$

When evaluating a multiclassification problem, it is common to decompose the multiclassification problem into a set of n 2-classification problems, each time using one of the classes as the positive class and the remaining classes uniformly as the negative class, and finally, calculating the average. *TP* indicates that the positive class is predicted to be positive, *TN* indicates that the negative class is predicted to be negative, *FP* indicates that the negative class is predicted to be positive, and *FN* indicates that the positive class is predicted to be negative.

### 4.2. Data Preprocessing

In this study, the P2020 component in the data processing module adopted fault injection to simulate the fault state, and all the multichannel data collected by fault injection were measured via voltage signal in V. The 16 collected channels were 1.05 V; 2.5 V; 3.3 V; 5 V supply voltage and ground voltage signals; 1.5 V of DDR3; the supply voltage of reference voltage 1 and reference voltage 2; 3.3 V and negative voltage of NOR FLASH; 3.3 V and negative voltage of NAND FLASH; 2.5 V; 3.3 V; and the reference voltage and negative voltage of P2020CPU on experimental circuit boards. Fourteen types of signal data were available for comparison with normal signals. The failure modes and collected parameters are shown in Table 1.

According to the avionics module, fault signals are generally characterized by large information entropy; we performed preliminary filtering by calculating the information entropy of the signals obtained from fault injection. If the information entropy of the fault signal was below the threshold level, we reviewed the fault injection process and eliminated the invalid data. The information entropy was calculated as follows:

$$H\left(X\right)=-\sum_{i=1}^{n}p\left(x_{i}\right)\log p\left(x_{i}\right)$$

where $p\left(x_{i}\right)$ denotes the probability that the random event X is $x_{i}$.

The simulation experiment is based on LabView’s data acquisition system software. According to the wiring to select the fault signal injection channel, we chose to parallel different capacitors or resistors to simulate different types of faults. For example, connecting a resistor to the P2020_CLK pin corresponds to the fault type of CPU processing capability degradation, and connecting a capacitor to the DDR3_CLK pin corresponds to the fault type of memory performance degradation. In order to realize fault injection and output waveform information acquisition, NI high-speed digital IO boards were used to provide excitation signals to the Field-Programmable Gate Array (FPGA). At the same time, the response signal of the FPGA was synchronously acquired back to the LabVIEW2012 software for data processing. Through AD conversion, the voltage, current, frequency and other parameters are stored in the specified format to the PC, thus completing the fault injection and data acquisition. The signal amplitude of the fault injection is small, and no filtering is set to avoid the effect of filtering noise reduction on the injected noise signal.

In deep learning tasks, data augmentation is the process of augmenting the size and diversity of the training dataset with a series of random transformations to increase the generalization ability and robustness of the model. Dataset enhancement is one of the most commonly employed methods of data enhancement; it must be noted that there are no specifically proposed dataset enhancement techniques for time series data; however, too-low data volumes available for training the model result in the risk of overfitting.

**Figure 7:**
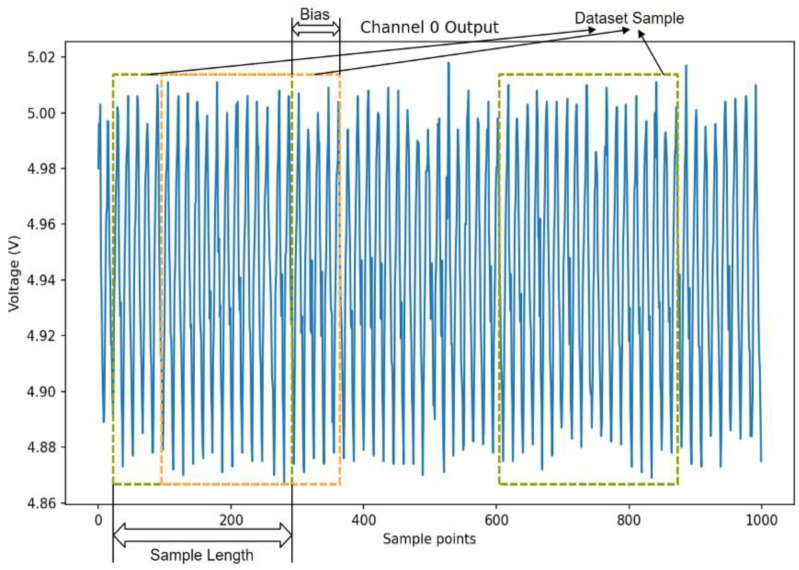
Overlapping sampling of samples.

In this study, overlapping sampling was taken to cut samples of length 256 in the original signal so that the training set contains a greater number of forms of samples and more feature information, increasing the diversity and adequacy of the data and providing additional information to support the training of the model. To control the overlapping area between different samples, this study set a suitable size of offset; in this case, it was set to 64 for samples with a length of 256. Ultimately, the generated dataset comprises 280,000 samples of 14 faults with 20,000 samples of each fault. For each fault type, 10,000 pieces of data were selected to divide the training set and validation set for Dataset1 in order to tune the hyperparameters. The rest of the data were divided into k folds for k-fold cross-validation, where k-1 folds were used as the training set and 1 fold as the test set, and the experiment was repeated k times, and k was chosen as 10 for this experiment. Since the model performed stably in Dataset1, the average of the evaluation metrics for each of the k experiments was used as the final test set result when k-fold cross-validation was performed in Dataset2. The data labels are in the one-hot form. One-hot coding converts the categorical variables into binary numeric vectors, where the vector index corresponding to the category is set to 1 at the index of the vector and 0 at other positions. The overlapping sampling method is shown in Figure 7. The multi-channel failure mode data set information is shown in Table 2.

### 4.3. Ablation and Alternative Experiment

The deep learning models presented in this study were implemented in pytorch1.11.0+cu113 and python3.9, and the configuration of the server used is NVIDIA GeForce RTX 4060-8GB. The model utilized the dataset for the fault dataset obtained from the sampling above, with a total of 14 fault types and 20,000 samples per class. The Dataset1 was utilized to determine the optimal hyperparameters, whose data were divided into a training set and validation set in the ratio of 8:2. Dataset2 was used for cross-validation by dividing it into 10 folds, arbitrarily taking 9 of them as the training set and the other as the test set and repeating the experiment 10 times.

The training was performed using a minimized cross-entropy loss function; for a multiclassification task, this can be expressed as the following:

$$Loss=-\sum_{i}\sum_{c=1}^{M}y_{ic}\log(p_{ic})$$

where *M* denotes the number of categories and $y_{ic}$ is a sign function (0 or 1), taking 1 if the true category of sample *i* is equal to c and 0 otherwise, and $p_{ic}$ is the predicted probability that observation sample *i* belongs to category c.

By obtaining the appropriate hyperparameters at Dataset1, in Dataset2 we made use of the Adam optimizer; this optimizer combines the Momentum and RMSprop gradient descent methods and proves to be efficiently applicable to different neural networks. Using the early-stopping mechanism, the epochs were set to 50 in the experiment, and the training was terminated when the validation set lost 7 epochs without decreasing. Training in batches was conducted using mini-batch gradient descent with the batch size set to 32. The initial learning rate was set to 0.0001, and the Reduce LR On Plateau was employed to dynamically adjust the learning rate by multiplying the learning rate by 0.1 times, the new learning rate when the validation set lost 2 epochs without degradation.

#### 4.3.1. AMTC Module Ablation Experiment

The ablation experiment is for the number of AMTC modules to be ablated. Where Model A makes no use of AMTC modules, Model B employs one AMTC module, Model C employs three AMTC modules, which is the model used in this study, and Model D employs five AMTC modules. Each AMTC module was connected using one residual connection to enhance the accuracy and stability of the model. The outcomes of model training with different numbers of AMTC module counts are shown in Table 3.

**Figure 8:**
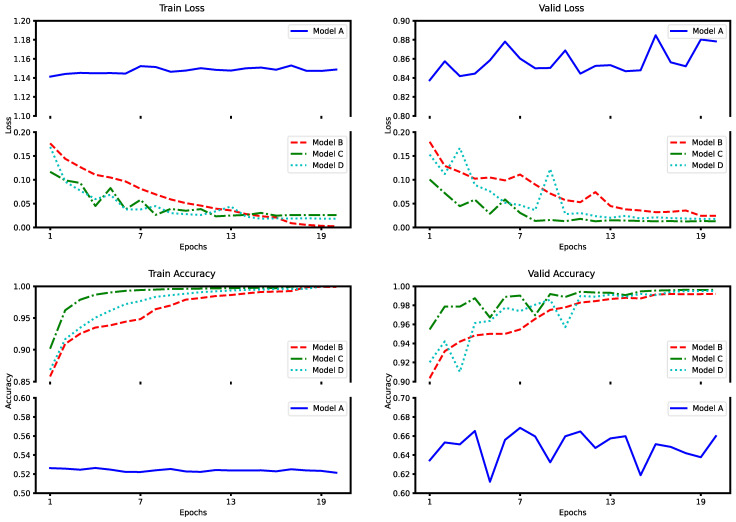
Training loss/accuracy curves and validation loss/accuracy curves for different number of AMTC modules on Dataset1.

The loss and accuracy curves obtained after training on Dataset1 are depicted in the Figure 8. 

The experiment data indicate that Model A has significantly low prediction accuracy and that the training results do not converge. Model B obtained a significant improvement in accuracy by using one AMTC module, indicating that the AMTC module has a strong feature extraction capability; however, due to its insufficient network depth, it has limited feature extraction ability; ultimately, it performs less efficiently than model C in the validation set and test set. The performance of Model D in the validation set fluctuates during training and does not converge as fast as Model C. Because Model D is overfitted and the gradient propagation is unstable, parameter optimization is difficult and it is more difficult to train. Compared to Models A, B, and D, Model C is found to be more accurate in the validation sets and test sets, as well as showing a steadier convergence process on the validation sets.

#### 4.3.2. Network Structure Ablation Experiment

For the network ablation experiments, the ICA module, the HBTA module, and the entire Hybrid Attention module were ablated, and the results after ablation are shown in Table 4.

**Figure 9:**
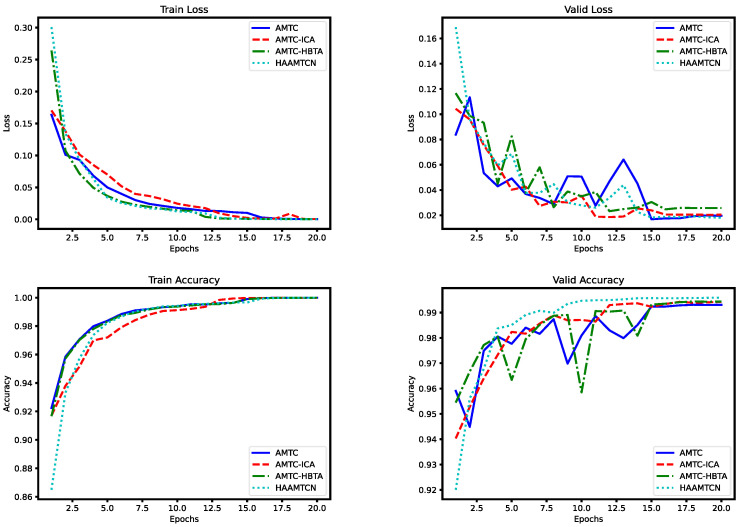
Training loss/accuracy curves and validation loss/accuracy curves before and after model ablation on Dataset1.

The loss curves and accuracy curves for the training set and validation set on Dataset1 are shown in the Figure 9.

The experimental data demonstrate that the removal of the ICA module and the HBTA module leads to a decrease in accuracy, while the convergence process is more unstable. The validity of the ICA module and the HBTA module is further illustrated by the fact that adding the ICA module allows the model to better focus on critical channel information, and the addition of the HBTA module facilitates the model to capture both the long-time dependencies as well as the proximity time step relationships. The two attention mechanisms are complementary since they pay attention to vital information from the channel dimension and time step dimension, which further optimizes the feature maps extracted by the AMTC module and raises the accuracy of the final fault classification.

#### 4.3.3. Network Module Replacement Experiment

Alternative experiments were conducted by replacing the attention module in this study with the SE Attention Block (Squeeze-and-Excitation Block), MulAtt (Multi-head Attention mechanism module), Cross-attention mechanism module, Gate module, and GCnet module (Global Correlation Network) for experimental comparison. The batch size and initial learning rate are also the same as in the above experiments, and the Adam optimizer was chosen to dynamically adjust the learning rate using ReduceLROnPlateau. The results of the experiment, after replacing it with other attention mechanisms, are shown in Table 5.

As shown by the experimental data, applying the SE bottleneck structure restricts the free flow of information in the network and is prone to overfitting. The Gate attention mechanism simply computes the attention weights and gating signals directly from the inputs without considering the interactions between the features. The multi-attention mechanism is hard to parameterize and it is difficult to capture the relationship between time steps through this mechanism. GCnet’s attention coefficients, computed by linear transformation and activation function, are fixed and have limited ability to model long-distance dependencies. The above attention mechanisms struggle to increase accuracy. The cross-attention mechanism that combines time-domain and frequency domain information shows some improvements in terms of model accuracy; however, the effect is relatively limited since avionics fault data do not have highly pronounced cyclic characteristics. Comparing the performance of the validation set and test set shows that the Hybrid Attention module adopted in this study achieves complementarity in the channel dimension and time step dimension. Thus, it is effective for avionics module fault diagnosis classification tasks by capturing inter-channel linkages in the channel dimension and long-time dependencies in the time step dimension.

### 4.4. Comparison Experiment

To conduct a comparison, a one-dimensional convolutional neural network was selected as the benchmark model in this paper, and DTW-KNN, SVM, TCN [23], MRes-FCN [24], MACNN [25], MLSTM-FCN [17], Inception-Resnet [26], and Inception-FCN [27] were selected as the comparison variables. The Adam optimizer was chosen for gradient descent, and ReduceLROnPlateau was employed to dynamically adjust the learning rate; the experimental results of this comparison are shown in Table 6.

**Figure 10:**
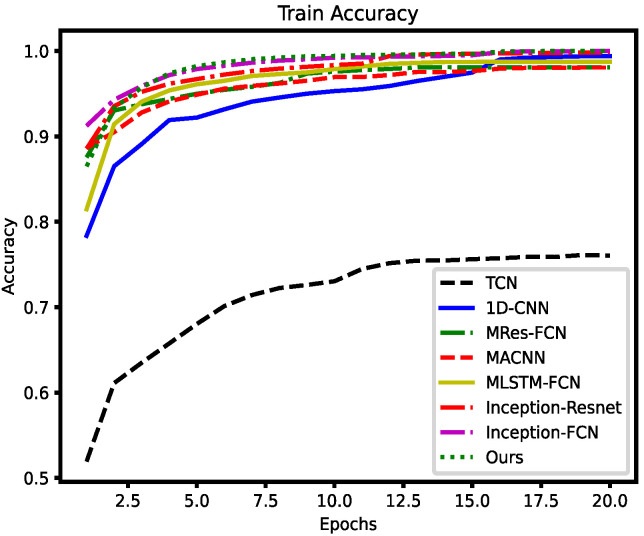
Training accuracy curves for different models on Dataset1.

**Figure 11:**
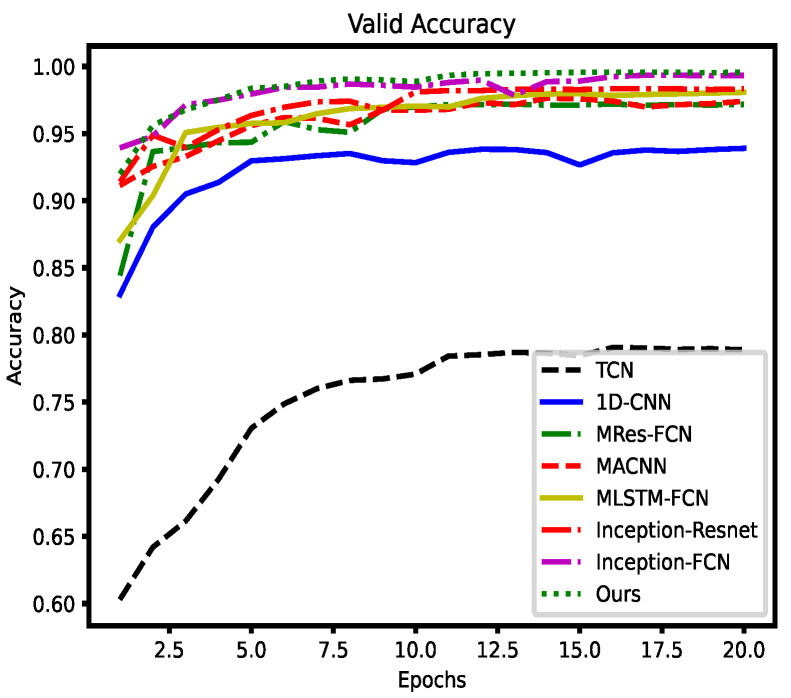
Validation accuracy curves for different models on Dataset1.

The accuracy curves obtained after training on Dataset1 are shown in the Figure 10 and Figure 11.

The experimental data indicate that the HAAMTCN achieves state-of-the-art results on avionics fault diagnosis tasks. The HAAMTCN achieves the highest accuracy on both the validation and test sets; it also achieves a fast convergence rate, relative to the benchmark model and other mainstream models for fault classification of temporal data. Thus, the results demonstrate that the HAAMTCN efficiently identifies the type of faults in avionics modules.

It is hard to set null rates of Temporal Convolutional Networks (TCN), which can lead to information loss or difficulty in training. A one-dimensional convolutional neural network as a baseline model, due to its limited feature extraction capability, is fast to train but falls short in terms of accuracy. Multi-scale residual full convolutional neural network (MRes-FCN) utilizes full convolutional block and residual block serial multi-scale convolutional kernel to extract multi-scale features; however, the network is deeper and slower to train, and feature extraction is not sufficiently adequate. Multi-scale Attention Convolutional Neural Network (MACNN) captures information at various scales along the timeline using only convolutional kernels of different sizes. Multivariate Long Short-Term Memory Fully Convolutional Network (MLSTM-FCN) consists of LSTM with convolutional blocks to extract features; however, its introduction of the SE module limits the correlation between channels and is prone to overfitting. The Inception module in Inception-Resnet has a simple structure with fewer layers and restricted feature extraction capability. The Inception-FCN combines multi-scale convolution as well as FCN to favorably capture temporal dependencies and sequence features in the data; however, the lack of an attentional mechanism makes it difficult to further improve accuracy. The HAAMTCN combines the advantages of Inception-Resnet and MACNN in extracting features from multiple scales while incorporating an attention mechanism to place greater emphasis on the vital information that contributes significantly to the improved accuracy rate. This demonstrates that the AMTC module has a stronger feature extraction capability, and that the attention mechanism enhances the model accuracy even further.

## 5. Conclusions

In this study, to address the lack of avionics module data acquisition difficulties and other issues, the fault injection method is adopted to simulate the different fault types of avionics modules. An overlapping sampling operation is adopted for the simulated avionics faults to construct the dataset for data augmentation, which facilitates the network model to extract features more adequately. The HAAMTCN for the integrated functional circuit module of the avionics module is proposed and is presented to diagnose different faults, which adaptively construct the optimal size of the convolutional kernel to efficiently extract features; further, the combined use of the Interaction Channel Attention (ICA) module and the Hierarchical Block Temporal Attention (HBTA) module results in the HAAMTCN to pay more attention to the critical information in the channel dimension and time step dimension. The advantages of HAAMTCN are verified through ablation experiments and comparative tests, which illustrate its effectiveness in avionics module fault diagnosis tasks and reflect its better ability to handle avionics module fault signals with large information entropy.

## Figures and Tables

**Table 1:** Multichannel acquisition data for injected fault signal.

Corresponding Failure Modes	Fault Injection Point	Injected Fault Signal Type
Normal	None	None
CPU processing power decreases	P2020_GND	Inject 0−0.2 V noise signal
	P2020_CLK	Inject 0−0.3 V 1 KHz square wave signal
		Parallel connection of 200 ohm resistors
		Parallel connection of 470 nF capacitors
Memory performance degradation	DDR3_GND	Inject 0−0.2 V noise signal
	DDR3_CLK	Inject 0−0.3 V 5 KHz square wave signal
		Parallel connection of 470 nF capacitors
		Parallel connection of 200 ohm resistors
	DDR3_CK2	Inject 0−0.3 V 5 KHz square wave signal
		Parallel connection of 200 ohm resistors
		Parallel connection of 470 nF capacitors
	NAND Flash_GND	Inject 0−0.2 V noise signal
	NOR Flash_GND	Inject 0−0.2 V noise signal

**Table 2:** Multichannel fault dataset.

Sample Size	Total Number of Samples	Number of Samples for Adjusting Hyperparameters (Datatset1)	Number of Samples for k-Fold Cross-Validation (Datatset2)	Fault Category
256 × 16	280,000	140,000	140,000	14

**Table 3:** Results for different number of AMTC modules.

Model	Number of AMTC Modules	Validation Set Accuracy (Dataset1)	Test Set Accuracy (Dataset2)	Test Set Precision (Dataset2)	Test Set Recall (Dataset2)	Test Set F1-Score (Dataset2)
A	0	65.99%	66.22%	66.10%	66.18%	66.14%
B	1	99.21%	99.32%	99.30%	99.30%	99.30%
C	3	99.59%	99.65%	99.62%	99.61%	99.62%
D	5	99.52%	99.44%	99.47%	99.44%	99.45%

**Table 4:** Results of the Hybrid Attention module ablation experiment.

Model	Validation Set Accuracy (Dataset1)	Test Set Accuracy (Dataset2)	Test Set Precision (Dataset2)	Test Set Recall (Dataset2)	Test Set F1-Score (Dataset2)
AMTC	99.30%	99.32%	99.36	99.36	99.36
AMTC-ICA	99.42%	99.44%	99.43	99.44	99.43
AMTC-HBTA	99.44%	99.54%	99.52	99.50	99.51
HAAMTCN	99.59%	99.65%	99.62%	99.61%	99.62%

**Table 5:** Results of the attention mechanism ablation experiment.

Attention Mechanism	Validation Set Accuracy (Dataset1)	Test Set Accuracy (Dataset2)	Test Set Precision (Dataset2)	Test Set Recall (Dataset2)	Test Set F1-Score (Dataset2)
SE	99.32%	99.30%	99.30%	99.32%	99.31%
MulAtt	99.33%	99.37%	99.35%	99.36%	99.35%
Gate	99.35%	99.28%	99.30%	99.28%	99.29%
GCnet	99.40%	99.40%	99.38%	99.38%	99.38%
FFT-CrossAttention	99.40%	99.42%	99.42%	99.45%	99.43%
ICA-HBTA	99.59%	99.65%	99.62%	99.61%	99.62%

**Table 6:** Results of different mainstream models.

Module	Validation Set Accuracy (Dataset1)	Test Set Accuracy (Dataset2)	Test Set Precision (Dataset2)	Test Set Recall (Dataset2)	Test Set F1-Score (Dataset2)
DTW-KNN	73.10%	71.36%	71.32%	70.55%	70.93%
TCN	78.96%	78.55%	77.88%	78.70%	78.29%
SVM	85.36%	84.20%	83.22%	82.85%	83.03%
1D-CNN	93.81%	93.65%	93.58%	93.58%	93.58%
MRes-FCN	97.19%	97.10%	96.81%	96.60%	96.70%
MACNN	97.40%	97.62%	97.55%	97.54%	97.53%
MLSTM-FCN	98.07%	98.26%	98.23%	98.04%	98.03%
Inception-Resnet	98.34%	98.50%	98.58%	98.58%	98.58%
Inception-FCN	99.33%	99.38%	99.39%	99.39%	99.39%
HAAMTCN	99.59%	99.65%	99.62%	99.61%	99.62%

## Data Availability

The data presented in this study are available on request from the corresponding author.
